# A High-Density Genetic Map for Soybean Based on Specific Length Amplified Fragment Sequencing

**DOI:** 10.1371/journal.pone.0104871

**Published:** 2014-08-12

**Authors:** Zhaoming Qi, Long Huang, Rongsheng Zhu, Dawei Xin, Chunyan Liu, Xue Han, Hongwei Jiang, Weiguo Hong, Guohua Hu, Hongkun Zheng, Qingshan Chen

**Affiliations:** 1 College of Agriculture, Northeast Agricultural University, Harbin, Heilongjiang, People's Republic of China; 2 The Crop Research and Breeding Center of Land-Reclamation of Heilongjiang Province, Harbin, Heilongjiang, People's Republic of China; 3 Biomarker Technologies Corporation, Beijing, China; Institute of Genetics and Developmental Biology, Chinese Academy of Sciences, China

## Abstract

Soybean is an important oil seed crop, but very few high-density genetic maps have been published for this species. Specific length amplified fragment sequencing (SLAF-seq) is a recently developed high-resolution strategy for large scale *de novo* discovery and genotyping of single nucleotide polymorphisms. SLAF-seq was employed in this study to obtain sufficient markers to construct a high-density genetic map for soybean. In total, 33.10 Gb of data containing 171,001,333 paired-end reads were obtained after preprocessing. The average sequencing depth was 42.29 in the Dongnong594, 56.63 in the Charleston, and 3.92 in each progeny. In total, 164,197 high-quality SLAFs were detected, of which 12,577 SLAFs were polymorphic, and 5,308 of the polymorphic markers met the requirements for use in constructing a genetic map. The final map included 5,308 markers on 20 linkage groups and was 2,655.68 cM in length, with an average distance of 0.5 cM between adjacent markers. To our knowledge, this map has the shortest average distance of adjacent markers for soybean. We report here a high-density genetic map for soybean. The map was constructed using a recombinant inbred line population and the SLAF-seq approach, which allowed the efficient development of a large number of polymorphic markers in a short time. Results of this study will not only provide a platform for gene/quantitative trait loci fine mapping, but will also serve as a reference for molecular breeding of soybean.

## Introduction

Soybean, *Glycine max* (L.) Merr., is not only a major protein source but also represents the world's leading oilseed crop, accounting for approximately 56% of global oilseed production [1]. Because of its economic importance and nutritional value, the study of soybean genetics and molecular biology has progressed rapidly in the past 20 years. For example, Keim et al. [2] constructed a soybean genetic map using restriction fragment length polymorphism (RFLP) markers, and Shoemaker et al. [3] and Lark et al. [4] simultaneously published molecular linkage maps. Cregan et al. [5] developed 606 soybean simple sequence repeat (SSR) loci for three mapping populations to create the first version of a soybean integrated genetic linkage map. Song et al. [6] generated a genetic linkage map by integrating five soybean genetic maps, including those for two F_2_ populations (‘A81-56022’×‘PI468916’ and ‘Clark’×‘Harosoy’) and for three recombinant inbred line (RIL) populations (‘Minsoy’×‘Noir1’, ‘Minsoy’×‘Archer’, and ‘Noir1’×‘Archer’). Choi et al. [7] constructed a soybean transcript map and analyzed the gene distribution, haplotypes, and single nucleotide polymorphisms (SNPs). With recent advances in genome sequencing, Hyten et al. [8], [9] built two high density integrated genetic linkage maps of soybean based genome sequencing and high-throughput SNP genotyping.

A genetic map provides an important foundation for quantitative trait loci (QTL) mapping, and the utility of genetic linkage maps depends on the types and numbers of polymorphic markers used. Next generation sequencing technology makes it possible to obtain thousands of SNPs throughout the genome that are potential markers for high-density genetic maps. Consequently, several cost effective methods for SNP discovery and high throughput genotyping were developed, such as RADseq [10] (restriction site-associated sequencing), double digest RADseq [11], and two-enzyme genotyping-by-sequencing (GBS) [12]. Recently, Sun et al. [13] developed specific length amplified fragment (SLAF) sequencing (SLAF-seq) as a high-resolution strategy for large-scale *de novo* SNP discovery and genotyping, this approach was successfully used to create a genetic map for common carp (*Cyprinus carpio* L.); it was the highest-density genetic map yet for any organism without a reference genome sequence. In this study, we used SLAF-seq to generate genotype data and subsequently constructed a high-density genetic map of soybean.

## Materials and Methods

### Plant material and DNA extraction

A F_21_ population of 147 RILs derived from a cross between ‘Charleston’ and ‘Dongnong594’. Seedlings of progeny and parents were planted in the experiment field of Northeast Agriculture University in Harbin (126°38′E, 45°45′N), Heilongjiang Province, China, in 2013. Young healthy leaves from the two parents and RIL individuals were collected and genomic DNA was extracted by the CTAB method [14]. DNA was quantified with an ND-1000 spectrophotometer (NanoDrop, Wilmington, DE, USA) and by electrophoresis in 0.8% agarose gels with a lambda DNA standard.

### Genotyping

SLAF-seq was used to genotype a total of 149 individuals, and the two parents, as previously described [13], with a few modifications. In brief, genomic DNA from each sample was treated with *Nde*I, *Mse*I (NEB, Ipswich, MA, USA), T4 DNA ligase (NEB), ATP (NEB), and *Mse*I adapter at 37 °C. These restriction-ligation reaction solutions were diluted and mixed with dNTP, Taq DNA polymerase (NEB) and *Mse*I primer containing barcode 1 for PCR reactions. The E.Z.N.A. Cycle Pure Kit (Omega, London, UK) were used to purify the PCR products. The purified PCR products were pooled and incubated at 37 °C with *Mse*I, T4 DNA ligase, ATP, and Solexa adapter. After incubation, the reaction products were then purified using a Quick Spin column (Qiagen, Venlo, Netherlands), and electrophoresed on a 2% agarose gel. SLAFs of 550–600 bp (including adapter sequence indexes and adaptors) in size were isolated using Gel Extraction Kits (Qiagen). These SLAFs were then subjected to PCR with Phusion Master Mix (NEB) and Solexa Amplification primer mix to add barcode 2. PCR products were gel purified and SLAFs of 330–380 bp selected for paired-end sequencing on an Illumina HiSeq 2500 sequencing platform (Illumina, San Diego, CA, USA). DNA sequence reads were 200 nucleotides long.

According to the barcode sequences, raw reads were demultiplexed to individuals. Then, low quality reads (quality score<20) were filtered out. After barcodes were trimmed from reads, reads of 100 bases from the same samples were mapped onto the soybean genome sequence [28] using SOAPdenovo2 software [15]. And the parameters r, M, m, and x were set as 0, 4, 50, and 1000, individually. Among them, r with 0 represents shielding repeats, M with 4 represents output the best comparison, m with 50 and x with 1 000 represent take insert size as 50 bp–1000 bp, for sequencing randomness. All sequence mapped to the same position were defined as a SLAF loci. In each of the SLAF, we found polymorphism locus between the parents, most of them are SNPs. All polymorphism SLAFs loci were genotyped with consistency in the offspring and parental SNP loci.

All SLAF markers have been four times filtered and quality assessment by the method described by Sun et al. [13]. A SLAF which has less than three SNP and average depths of each sample above 3, was used as a high quality SLAF markers. The markers with parental homozygous were used to construct high-density genetic map.

### Linkage map construction

All high quality of SLAFs markers were allocated into 20 linkage groups (LGs) based on their locations on chromosomes. Considering that Next generation sequencing (NGS) data may cause many genotyping errors and deletion, which can greatly reduce the quality of high-density linkage maps, High Map Strategy was used to order SLAF markers and correct genotyping errors within LGs [16]. Detaily MSTmap algorithm was used to order SLAFs markers [17] and the SMOOTH algorithm [18] was used to correct genotyping errors following marker ordering. All linkage groups have undergone these procedures: we firstly get a primary marker orders by their location on chromosomes, according to relationship between ordered markers, genotyping errors or deletion were corrected by SMOOTH algorithm, after that we use MSTmap to order the map, again we took SMOOTH to corrected the new ordered genotypes. As 4 or more cycles, we have 20 high-quality maps. Map distances were estimated using the Kosambi mapping function [19].

## Results

### Analysis of SLAF-seq data and SLAF markers

**Figure 1:**
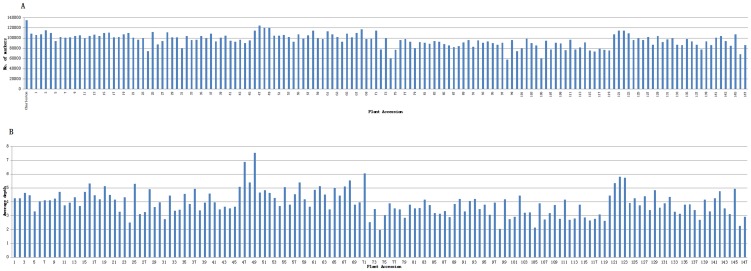
Number of markers and average sequencing depth. The x-axes in A and B indicate individual recombinant inbred line plant accessions, and the y-axes indicate the number of markers (A) and average depth (B).

**Figure 2:**
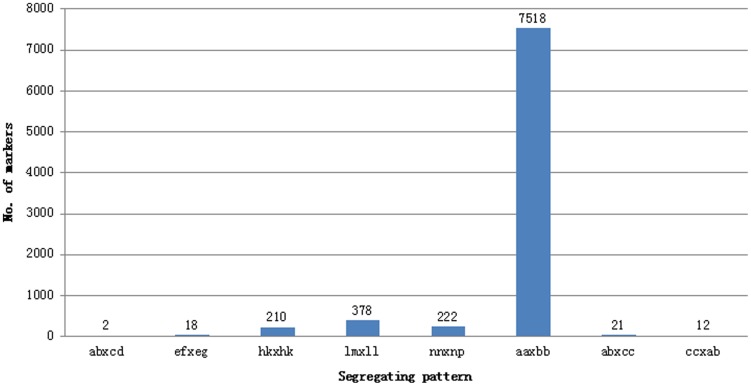
Number of markers in each of eight segregation patterns.

DNA sequencing generated a total of 33.10 Gb of raw data, consisting of 171,001,333 paired-end reads of ∼100 bp in length left over after preprocessing (Data has been submitted to DNA Data Bank of Japan, the submission ID was DRA002239). Among them, 81.06% bases were of high-quality, with quality scores of at least 20 (Q20, indicating a 1% chance of an error, and thus 99% confidence). In total, 79 M reads after shielding repeats were accuracy paired-ends mapping to soybean reference genome,which paired ends mapping ratio were 46%. After that 164,197 SLAFs were detected of which the average sequencing depth was 42.29 in the Dongnong594, 56.63 in the Charleston, and 3.92 in each progeny (Figure 1). Among them 12,577 of which were polymorphic, giving a polymorphism rate of only 7.66%. The number of SLAF markers per chromosome ranged from 234 to 1,055 (Table 1). After filtering out the SLAFs lacking parent information, 8,381 were obtained and classified into eight segregation patterns (Figure 2). After filtered low quality SLAF markers and markers with heterozygous parents, 5,308 markers were high quality suitable to construct a linkage map for RIL population, with the criteria of segregation distortion (P<0.05) and the integrity of individual segregation patterns more than 70%. Average sequencing depths of these 5,308 markers were 79.33-fold in the Dongnong594, 48.54-fold in the Charleston, and 3.79-fold in each RIL individual (Table 2).

**Table 1:** SLAF markers numbers on each chromosome.

Chromosome ID	SLAF number	Polymorphism SLAF
Gm01	10,579	620
Gm02	9,067	704
Gm03	7,831	915
Gm04	8,932	378
Gm05	7,557	361
Gm06	8,674	766
Gm07	7,552	546
Gm08	7,814	334
Gm09	7,977	403
Gm10	8,820	722
Gm11	6,729	234
Gm12	6,901	249
Gm13	7,096	667
Gm14	9,000	720
Gm15	8,854	1,055
Gm16	6,601	517
Gm17	6,889	978
Gm18	9,954	1,001
Gm19	9,003	975
Gm20	8,367	432
Total	164,197	12,577
Total Depth	68,768,249	5,346,410

**Table 2:** Summary of marker depths.

Samples	Marker number	Total depth	Average depth
Charleston	5308	421083	79.33
Dongnong594	5308	257652	48.54
Average of Offspring	5308	20122	3.79

### Basic characteristics of the genetic map

In total, 5,308 markers were assigned to 20 LGs, including the SLAF markers physical positions (Table S1). In total, 5308 genomic regions were covered The LGs were numbered according to the chromosome numbers. The map spanned a total of 2294.433 cM with an average inter-marker distance of 0.43 cM (Table 3, Figure S1). On average, one LG contained 265 markers that spanned an average of 114.72 cM. The genetic length of the LGs ranged from 53.11 cM (Gm11) to 194.87 cM (Gm17). The LGs were numbered according to the chromosome numbers. Gm14 was the most saturated, having 330 markers with an average marker density of 0.29 cM, whereas Gm20 had the largest average inter-marker distance of 0.69 cM. The largest LG, Gm19, harbored 457 markers, covering a length of 186.902 cM with only 0.41 cM average inter-marker distance. The smallest LG, Gm11, contained 95 markers, with a length of 53.11 cM and an average inter-marker distance of 0.56 cM.

**Table 3:** Description on basic characteristics of the 20 linkage groups.

Linkage group ID	Marker number	Total distance(cM)	Average distance(cM)	Gaps≤5
Gm01	261	78.844	0.30	100%
Gm02	317	153.368	0.48	99.68%
Gm03	359	118.204	0.33	100%
Gm04	152	70.528	0.46	100%
Gm05	154	94.011	0.61	99.35%
Gm06	328	105.975	0.325	100%
Gm07	213	84.139	0.40	100%
Gm08	129	80.118	0.62	100%
Gm09	144	84.916	0.59	100%
Gm10	335	102.498	0.31	100%
Gm11	95	53.108	0.56	100%
Gm12	109	69.077	0.63	99.07%
Gm13	267	146.321	0.55	98.87%
Gm14	330	94.436	0.29	99.39%
Gm15	437	170.72	0.39	100%
Gm16	197	95.935	0.49	100%
Gm17	427	194.87	0.46	100%
Gm18	406	178.93	0.44	100%
Gm19	457	186.902	0.41	100%
Gm20	191	131.533	0.69	100%
Max linkage group	427	194.87	0.46	100%
Min linkage group	95	53.108	0.56	100%
Total	5,308	2,294.43	0.43	99.84%

‘Gap≤5’ indicated the percentages of gaps in which the distance between adjacent markers was smaller than 5 cM.

### Visualization and evaluation of the genetic map

Haplotype maps and heat map were used to evaluation the quality of genetic map. Haplotype map reflects the population of double crossover, suggested the genotyping errors. Haplotype maps were generated for each of the 147 RILs and for the parental controls using 5,308 SLAF markers (Figure S2) as described by West et al. [20]. Haplotype maps intuitively displayed the recombination events of each individual (Figure S2). Most of the recombination blocks were clearly defined. Less than 0.1% had heterozygous fragments, and less than 0.6% was missing. Although high frequency recombination events in RILs, all linkage groups distribute uniformity, only a few heterozygosity sites exist. Therefore, the RIL populations were well purified and suitable for genetic analysis.

Heat map reflects the relationship of recombination between markers from one single linkage group, the reaction was used to find ordering errors. Heat maps were also created to evaluate the genetic map quality by using pair-wise recombination values for the 5,308 SLAF markers (Figure S3). Most of the LGs performed well in visualization in general.

## Discussion

### Feasibility and advantages of SLAF sequencing for developing markers

SLAF-seq technology is highly automated because it was developed using bioinformatics for high-throughput sequencing technology applications. Differences between RAD-seq,SLAF is measured by sequencing the paired-ends of the sequence-specific restriction fragment length, RAD sequence is a sequence of measured randomly interrupted after the restriction sites surrounding. Since fragment length selection and not through random interrupted, SLAF repeatability is better than the RAD. It can generate large amounts of sequence information and handle whole genome density distributions. In contrast to inefficient, expensive, and time-consuming conventional methods of developing markers [21]–[23], SLAF sequencing ensured the density, uniformity, and efficiency of marker development. Since SLAF-seq methods were first developed, they have been used in several studies; for example, Zhang et al. [24] constructed the first high-density genetic map for sesame, Huang et al. [25] reported a draft genome of the kiwifruit *Actinidia chinensis*, and Chen et al. [26] studied the development of 7E chromosome-specific molecular markers for *Thinopyrum elongatum*. In this study, we developed markers on a large scale for soybean. We generated 33.10 Gb of raw data, consisting of 171,001,333 paired-end reads of ∼100 bp in length, including 164,197 high-quality SLAF markers with a polymorphism rate of only 7.66%. The data quantity was sufficient for marker development; as in recent research, the SLAF-seq method allowed the development of large numbers of highly accurate markers, making it especially suited for analyzing species with low polymorphism rates [24]. Although the polymorphism rate was low, the number of SLAF markers covered all soybean chromosomes, which had from 234 to 1,055 polymorphic markers each, and a total of 5,308 polymorphic markers were identified for genetic linkage map construction. Marker integrity and accuracy were high and marker quality and quantity met the requirements for construction of a genetic map. Therefore, SLAF-seq technology is ideal for developing plant chromosome-specific molecular markers with high success rates, specificity, and stability at low cost.

Due to the difference between the reference genome and the parent genome, MSTmap row map algorithm does not reference numerals genome sequence, the presence of the soybean genome a lot of transposon presence sequential order and genetic map of the reference genome many SLAF mark certain differences, such as Gm19.

### Future applications for the genetic map

To our knowledge, the high density genetic map reported in this paper had the smallest average distance (0.43 cM) between adjacent markers for soybean, although even this is not saturated enough. The latest version of the soybean genetic map was reported by Hyten et al. [8], [9] and created an integrated genetic linkage map of 5,500 markers spanning a genomic map distance of 2296.4 cM in five populations using the GoldenGate assay SNP detection system (Illumina), with a mean genetic distance of 0.6 cM between any consecutive pair of mapped SNP markers. In this study, we report a map with 5,308 markers on 20 LGs in one RIL population using SLAF-seq. The map spans 2,294.433 cM, with an average distance of 0.43 cM between adjacent markers. Although some SLAF markers showed at the same position on this genetic map, actually, the physical position was not at the same location (Table S1). And these markers could be used for different population, may show different diversity. Visual evaluation of the genetic map was performed using haplotype maps and heat maps and demonstrated that the RIL populations were well purified and suitable for genetic analysis. This map used parents from the USA and China, which could increase genetic diversity in the progeny, compared with the previous version of the soybean consensus map. And the validation of correlation of the genetic and physical positions shows good in this genetic map (Figure S4), the correlation should be the very important evaluation of the genetic map described by Sim et al. (2012), who developed a large SNP genotyping array and generated a genetic map using the SNP array and tomato RIL populations, the correlation of the genetic and physical positions shows very good [27].This map was also constructed using a single population, allowing us to easily find important RIL populations after gene/QTL fine mapping and gene cloning. Furthermore, the average distance between adjacent markers was 0.43 cM, the smallest average distance yet reported for soybean. Finally, SLAF-seq technology allowed a high success rate and excellent specificity and stability at low cost. As soybean genome research has advanced in recent years, Schmutz et al. [28] researched the genome sequence of the palaeopolyploid soybean and analyzed the genome structure, gene composition and repetitive DNA, and whole-genome duplication events. Lam et al. [29] reported a large-scale analysis of the patterns of genome-wide genetic variation in soybeans in which they re-sequenced and compared 17 wild and 14 cultivated soybean genomes and identified higher allelic diversity in wild soybean. In this study, 149 RILs populations and their parents were sequenced by the SLAF-seq method, and a high-density genetic map was constructed. Furthermore, additional data were generated during the sequencing process. In the figure, gene annotations and other information should be mined and combined with our high-density genetic map and the results of previous research, because our map was constructed based on molecular markers developed at the whole genome level. This high-density genetic map will provide a foundation for further research in fine mapping gene/QTL and molecular breeding.

## Supporting Information

Figure S1 **Soybean high density genetic map.** The map was constructed based SLAF markers and markers position in table S1.
(RAR)

Figure S2 **Haplotype map of the genetic map.** Green represents Dongnong594, blue represents Charleston, white means the parent could not be estimated, gray represents deletions, and red indicates heterozygosity.
(RAR)

Figure S3 **Heat map of the genetic map.** Each cell represents the recombination rate of two markers. Yellow indicates a lower recombination rate and purple a higher one.
(RAR)

Figure S4 **The correlation of the genetic and physical positions.** Axis of abscissa represents the genetic linkage group, axis of ordinate represents the physical positions.
(RAR)

Table S1 **The SLAF markers genotype, genetic linkage group and SLAF markers physical position.**
(XLSX)
