# Correction: A High-Density Genetic Map for Soybean Based on Specific Length Amplified Fragment Sequencing

**DOI:** 10.1371/journal.pone.0114349

**Published:** 2014-11-25

**Authors:** The PLOS ONE Staff

There are errors in Table 3. The values in the Linkage group ID and Total distance (cM) columns of the Max linkage group row and the values in the Total distance (cM) and Average distance (cM) columns of the Min linkage group row are incorrect. The authors have provided the corrected table below.

**Table 3:** Description on basic characteristics of the 20 linkage groups.

Linkage group ID	Marker number	Total distance(cM)	Average distance(cM)	Gaps≤5
Gm01	261	78.844	0.30	100%
Gm02	317	153.368	0.48	99.68%
Gm03	359	118.204	0.33	100%
Gm04	152	70.528	0.46	100%
Gm05	154	94.011	0.61	99.35%
Gm06	328	105.975	0.325	100%
Gm07	213	84.139	0.40	100%
Gm08	129	80.118	0.62	100%
Gm09	144	84.916	0.59	100%
Gm10	335	102.498	0.31	100%
Gm11	95	53.108	0.56	100%
Gm12	109	69.077	0.63	99.07%
Gm13	267	146.321	0.55	98.87%
Gm14	330	94.436	0.29	99.39%
Gm15	437	170.72	0.39	100%
Gm16	197	95.935	0.49	100%
Gm17	427	194.87	0.46	100%
Gm18	406	178.93	0.44	100%
Gm19	457	186.902	0.41	100%
Gm20	191	131.533	0.69	100%
Max linkage group	457	194.87	0.69	100%
Min linkage group	95	53.108	0.29	98.87%
Total	5,308	2,294.43	0.43	99.84%

‘Gap ≤ 5’ indicated the percentages of gaps in which the distance between adjacent markers was smaller than 5 cM.
